# The U-shaped association of non-high-density lipoprotein cholesterol with all-cause and cardiovascular mortality in general adult population

**DOI:** 10.3389/fcvm.2023.1065750

**Published:** 2023-02-08

**Authors:** Yu Huang, Meng Qi Yan, Dan Zhou, Chao Lei Chen, Ying Qing Feng

**Affiliations:** ^1^School of Medicine, South China University of Technology, Guangzhou, China; ^2^Department of Cardiology, Guangdong Provincial People’s Hospital (Guangdong Academy of Medical Sciences), Southern Medical University, Guangzhou, China

**Keywords:** non-high-density lipoprotein cholesterol, all-cause mortality, cardiovascular mortality, adult population, U-shaped

## Abstract

**Background:**

Non-high-density lipoprotein cholesterol (non-HDL-C) has been associated with atherosclerosis. However, the association between non-HDL-C and mortality in adult population remains unclear. We intended to investigate the association of non-HDL-C with cardiovascular and all-cause mortality using national representative data.

**Methods:**

The study included 32,405 participants from the National Health and Nutrition Examination Survey (1999–2014). Mortality outcomes were ascertained by linkage to National Death Index records through December 31, 2015. Multivariable-adjusted Cox regression models were used to evaluate hazard ratio (HR) and 95% confidence interval (CI) of non-HDL-C concentrations in quintiles. Two-piecewise linear regression and restricted cubic spline analyzes were performed to test dose–response associations.

**Results:**

After a median follow-up of 98.40  months, 2,859 (8.82%) all-cause and 551 (1.70%) cardiovascular deaths occurred. Compared with the highest group, the multivariable-adjusted hazard ratio (HR) of the first quintile for all-cause mortality was 1.53 (95%CI, 1.35–1.74). Higher non-HDL-C above a cutoff value of 4.9 mmol/L was related with cardiovascular mortality (HR = 1.33, 95%CI, 1.13–1.57). A U-shaped relationship between non-HDL-C and all-cause mortality was found in spline analysis with a cutoff value around 4 mmol/L. Similar results in subgroups analyzes were found among male, non-white population, participants who were not taking lipid-lowering drugs, and with body mass index (BMI) <25 kg/m^2^.

**Conclusion:**

Our findings suggest a U-shaped association between non-HDL-C and mortality among adult population.

## Introduction

Among atherogenic lipoproteins, non-high-density lipoprotein cholesterol (non-HDL-C) is reported to be the major contributor to atherosclerosis and the progression of cardiovascular disease (CVD) (1). Non-HDL-C is also more accurate than conventional lipid measurements at predicting clinical outcomes (2). Previous studies have well-established the relationship between higher non-HDL-C and CVD risk factors such as carotid plaques and diabetes (3, 4). However, few studies had discussed about the association between non-HDL-C and mortality, especially in general population. A recent study found an inverse association between serum non-HDL-C levels and mortality in patients undergoing hemodialysis (5). A recent study showed a U-shaped relationship between non-HDL-C and the risk of overall and cardiovascular mortality in patients with chronic kidney disease stage 3–5 (6). However, there was no association detected between non-HDL-C and all-cause mortality in studies on Mediterranean populations and elderly European males (7, 8). Therefore, the relationship between non-HDL-C and mortality in the general population remains controversial and unclear. Hence, the objective of this study was to investigate associations of non-HDL-C with all-cause and cardiovascular mortality in a large cohort study of general adult population.

## Methods

### Study population

**Figure 1:**
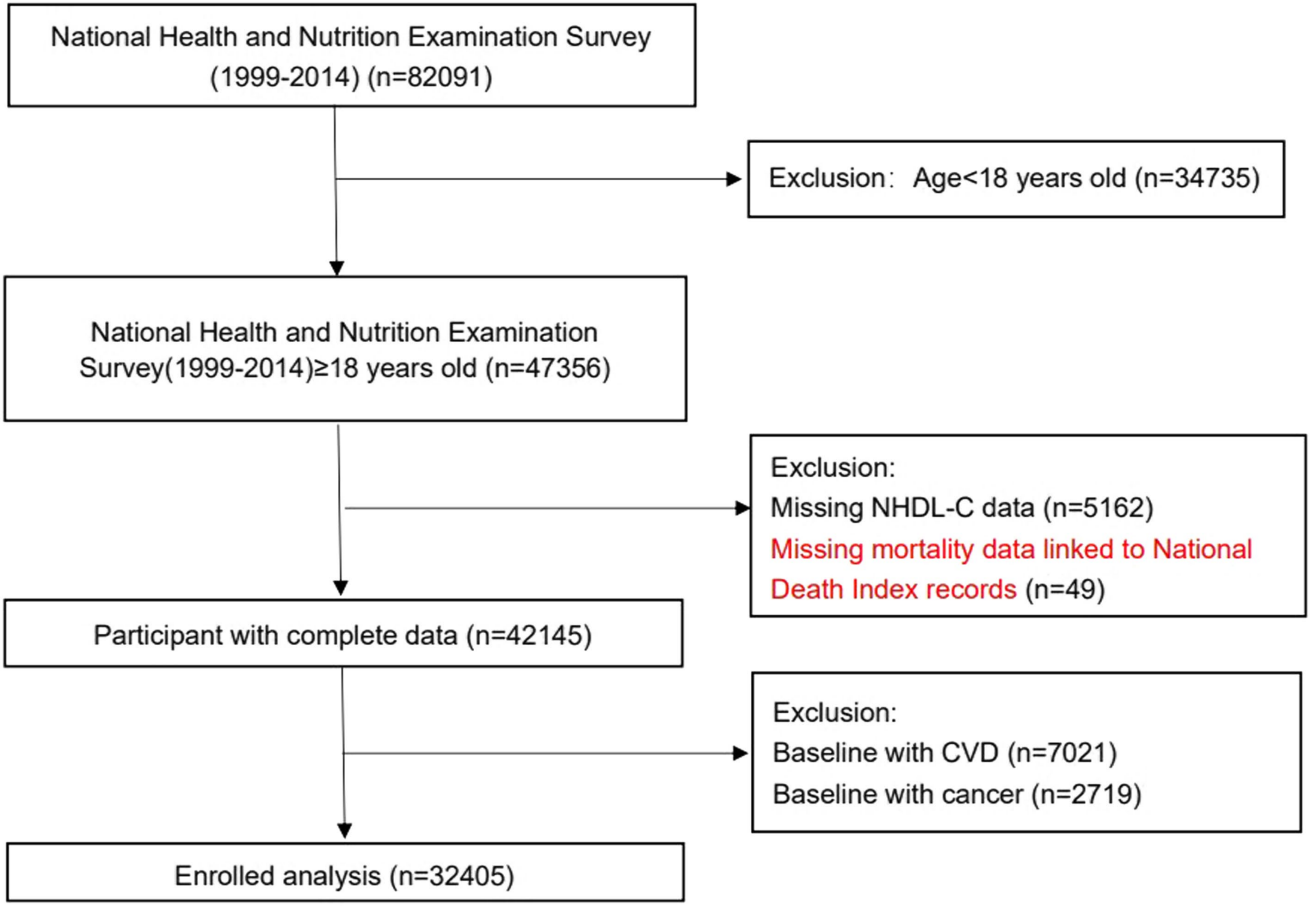
Study cohort.

Data were extracted from National Health and Nutrition Examination Surveys (NHANES) between 1999 and 2014, which was a stratified, multistage, nationally representative survey launched by the National Center for Health Statistics of the Center for Disease Control and Prevention in the United States. The NHANES is widely used by linking it to the National Death Index database. Approval of the survey protocol was obtained from the Institutional Review Board of the Centers for Disease Control and Prevention. All participants have provided written informed consent. In this study, participants older than 18 years old with non-HDL-C data were selected (*n* = 82,091). As shown in Figure 1, we excluded those aged <18 years old (*n* = 34,735), missing non-HDL-C (*n* = 5,162), people with CVD (*n* = 7,021) or cancer (*n* = 2,719) at baseline, and those lost to follow-up (*n* = 49). Finally, 32,405 subjects were included.

### Lipid measurements

A detailed description of the specific laboratory measurements can also be found on the official website at https://wwwn.cdc.gov/nchs/nhanes. To summarize, blood samples were collected in a fasting state by trained personnel to measure serum lipids (9): total cholesterol (TC), high-density lipoprotein cholesterol (HDL-C) (10), triglycerides (TG). Low density lipoprotein cholesterol (LDL-C) was computed by the Friedewald formula when TG ≤ 400 mg/dL (11). The level of serum non-HDL-C was calculated by TC minus HDL-C.

### Outcomes

Mortality data were extracted from the public-use linked mortality files, which included a set of mortality variables for adult participants only. National Death Index (NDI) captured vital status and cause of death of survey participants through December 31, 2015. The follow-up period was calculated from the date of lipid measurements until death or the end of the follow-up, whichever occurred first. We defined deaths from all causes as all-cause mortality. Cardiovascular mortality included deaths caused by CVD or cerebrovascular diseases (ICD-10 codes I00 to I09, I11, I13, I20 to I51, and I60 to I69) according to International Classification of Diseases, 10th Edition (ICD-10).

### Covariates

Questionnaires and examinations in NHANES were conducted according to standardized methods. Sociodemographic data (age, sex, race, family income, education level), lifestyle and behaviors (smoking and drinking status, physical activity), comorbidities (hypertension and diabetes), and current medication usage (hypoglycaemic, antihypertensive, and lipid-lowering drugs) were collected ad baseline data. As part of the physical examination, the patient’s height, weight, systolic blood pressure (SBP), and diastolic blood pressure (DBP) were measured. As defined by the World Health Organization, the body mass index (BMI) is the product of weight in kilograms multiplied by the square of height in meters. Calculations of estimated glomerular filtration rate (eGFR) were performed using the Modification of Diet in Renal Disease formula. Hypertension was defined as SBP ≥140 mmHg, DBP ≥90 mmHg, taking antihypertensive medications, or self-reported history of hypertension diagnosed by physicians (12). Diabetes mellitus was defined as FBG ≥ 126 mg/dL, self-report, hemoglobin A1c(HbA1C) ≥6.5%, or using hypoglycaemic drugs (13).

### Statistical analyzes

To account for the complex survey design of NHANES, appropriate sampling weights were used to reconstitute data on the US non-institutionalized population. Participants were divided into quintile groups by the non-HDL-C level. Variables were marked by mean values with standard deviation (SD) or percentages, as appropriate. The linear trend for baseline characteristics was tested by linear or logistic regression whenever appropriate. Cox proportional hazards regression models were applied to estimate hazard ratios (HRs) with 95% confidence intervals (95% CIs) for mortality according to the non-HDL-C quintiles, using the lowest quintile (Q1) as the reference group. Three models were fitted for Cox regression. Model I did not account for any covariates. Model II was adjusted for age, sex, and race. Model III was additionally adjusted for smoking, family income, education level, physical activity, BMI, SBP, diabetes, and use of antihypertensive drugs, hypoglycemic agents, and lipid-lowering drugs. Restricted cubic spline (RCS) analysis was used to identify a cut-off point of non-HDL-C for mortality. Further the two piecewise regression analysis was performed by stratifying non-HDL-C level into a binary variable at the selected cut-off point. Logarithmic likelihood ratio tests were conducted to compare the differences between the two piecewise linear regression models. Finally, we performed subgroup analyzes by age, sex, race, lipid-lowering drug usage, BMI, physical activity, education level, and family income. All statistical analyzes were performed using R version 3.6.3 (R Foundation for Statistical Computing, Vienna, Austria), with *p* < 0.05 confirmed as statistical significance.

## Results

### Baseline characteristics

Table 1 described the baseline of 32,405 participants with an average age of 46.13 ± 17.26 years and 15,378 (47.46%) were men. In terms of non-HDL-C concentrations, individuals in the upper non-HDL-C quintiles tended to be older, male, and have higher rates of smoking and hypertension. Moderate activity ratio and mortality risk tended to be higher with the increase of non-HDL-C while education level and vigorous activity ratio were decreased with increasing non-HDL-C quintiles (all *p* for trend <0.01). For the median follow-up of 98.40 months, 2,859 (8.82%) all-cause and 551 (1.70%) cardiovascular deaths occurred. All-cause and cardiovascular mortality differed significantly across non-HDL-C quintiles (*p* < 0.05).

**Table 1:** Demographic and clinical characteristics according to non-HDL-C quintiles.

	Total	Non-HDL-C					*p* for trend
		Q1	Q2	Q3	Q4	Q5	
Age, years	43.99 (0.18)	39.08 (0.33)	42.66 (0.27)	44.66 (0.25)	46.31 (0.25)	47.29 (0.24)	<0.01
Female, %	51.63 (0.27)	57.78 (0.78)	56.49 (0.80)	51.77 (0.78)	47.87 (0.70)	44.01 (0.82)	<0.01
White, %	67.69 (1.19)	64.00 (1.33)	66.75 (1.35)	68.70 (1.34)	68.83 (1.31)	70.21 (1.33)	<0.01
Smoking, %	45.23 (0.57)	41.84 (0.92)	43.41 (0.88)	44.42 (0.83)	45.21 (0.86)	51.41 (0.96)	<0.01
Education level, %							
High school or above	82.01 (0.49)	83.19 (0.68)	83.07 (0.69)	82.70 (0.68)	81.27 (0.69)	79.75 (0.65)	<0.01
Family income	3 (0.03)	2.88 (0.04)	2.98 (0.04)	3.08 (0.04)	3.10 (0.04)	2.95 (0.04)	0.01
Physical activity, %							
Moderate activity	28.27 (0.46)	25.61 (0.76)	27.09 (0.72)	28.80 (0.76)	29.40 (0.82)	30.50 (0.84)	<0.01
Vigorous activity	31.6 (0.63)	38.03 (1.09)	33.72 (0.91)	30.34 (0.88)	29.93 (0.95)	25.87 (0.97)	<0.01
Body mass index, kg/m^2^	28.42 (0.07)	26.35 (0.12)	27.85 (0.12)	28.94 (0.11)	29.21 (0.10)	29.79 (0.11)	<0.01
Systolic blood pressure, mmHg	121.12 (0.18)	116.96 (0.28)	119.40 (0.24)	121.35 (0.31)	122.83 (0.27)	125.11 (0.33)	<0.01
Diastolic blood pressure, mmHg	71.14 (0.16)	67.70 (0.23)	69.93 (0.22)	71.25 (0.21)	72.64 (0.22)	74.21 (0.25)	<0.01
eGFR, mg/min/1.73m^2^	88.71 (0.32)	91.90 (0.52)	89.61 (0.44)	88.45 (0.60)	86.99 (0.46)	86.53 (0.42)	<0.01
Total cholesterol, mmol/L	5.13 (0.01)	3.89 (0.01)	4.58 (0.01)	5.04 (0.01)	5.57 (0.01)	6.61 (0.02)	<0.01
HDL cholesterol, mmol/L	1.37 (0)	1.50 (0.01)	1.44 (0.01)	1.36 (0.01)	1.31 (0.01)	1.22 (0.01)	<0.01
LDL cholesterol, mmol/L	3.04 (0.01)	1.97 (0.01)	2.60 (0.01)	3.03 (0.01)	3.51 (0.01)	4.39 (0.02)	<0.01
Triglycerides, mmol/L	1.53 (0.02)	0.91 (0.01)	1.20 (0.02)	1.41 (0.02)	1.70 (0.02)	2.57 (0.06)	<0.01
Non-HDL-C, mmol/L	3.76 (0.01)	2.39 (0.01)	3.14 (0.01)	3.67 (0.01)	4.26 (0.01)	5.39 (0.01)	<0.01
Comorbidities, %							
Hypertension	33.22 (0.44)	25.70 (0.74)	31.15 (0.70)	34.23 (0.84)	35.16 (0.89)	39.98 (0.84)	<0.01
Diabetes	10.04 (0.23)	10.06 (0.49)	10.06 (0.46)	9.03 (0.47)	9.45 (0.40)	11.62 (0.48)	0.12
Treatment, %							
Antihypertensive drugs	17.67 (0.35)	15.38 (0.62)	18.07 (0.51)	19.00 (0.62)	17.94 (0.69)	17.93 (0.62)	<0.01
Hypoglycemic agents	5.09 (0.16)	6.56 (0.39)	5.89 (0.36)	4.18 (0.31)	4.11 (0.32)	4.68 (0.36)	<0.01
Lipid-lowering drugs	8.81 (0.25)	11.03 (0.52)	11.56 (0.61)	9.01 (0.51)	6.71 (0.38)	5.63 (0.43)	<0.01
Outcomes, %							
Cardiovascular disease mortality	1.04 (0.06)	0.72 (0.10)	0.88 (0.10)	0.97 (0.13)	1.30 (0.14)	1.37 (0.14)	<0.01
All-cause mortality	6.12 (0.19)	5.35 (0.30)	5.19 (0.32)	5.92 (0.29)	6.58 (0.33)	7.59 (0.41)	<0.01

Q, quintiles; *n*, number; eGFR, estimated glomerular filtration rate; HDL-C, high density lipoprotein cholesterol; LDL-C, low density lipoprotein cholesterol; non-HDL-C, non-high-density lipoprotein cholesterol. Values are mean or percent with standard error. *p* for trend was tested by linear or logistic regression.

### Hazard ratios for all-cause and cardiovascular mortality

Non-HDL-C, as a continuous variable (per 1 mmol/L increase), after fully adjusted (model III), was found to be independently related with decreased all-cause mortality (HR = 0.93, 95%CI, 0.88–0.99), but not cardiovascular mortality (HR = 1.08, 95%CI, 0.96–1.22) (Table 2). When using the first quintile of non-HDL-C (Q1) as reference, the multivariable-adjusted HRs (95% CI) of Q2 to Q5 for all-cause mortality were 0.74 (0.63–0.87), 0.73 (0.63–0.84), 0.66 (0.56–0.78), and 0.68 (0.58–0.79), respectively (*p* for trend<0.01), and for cardiovascular mortality were 1.00 (0.72–1.40), 0.98 (0.70–1.39), 1.12 (0.76–1.65), and 1.08 (0.75–1.55) compared to Q1, respectively (*p* for trend = 0.56). Details are displayed in Table 2.

**Table 2:** Multivariate cox regression analysis of non-HDL-C with cause-specific mortality.

	Event rate/1,000 person-years	Model I HR (95%CI)	Model II HR (95%CI)	Model III HR (95%CI)
All-cause mortality				
Non-HDL-C (as continuous variables, mmol/L)	7.20	1.1 (1.05, 1.15)	0.96 (0.90, 1.01)	0.93 (0.88, 0.99)
Non-HDL-C quintiles				
Q1	6.77	Reference	Reference	Reference
Q2	6.25	0.91 (0.77, 1.08)	0.71 (0.61, 0.84)	0.74 (0.63, 0.87)
Q3	7.03	1.03 (0.89, 1.18)	0.70 (0.60, 0.81)	0.73 (0.63, 0.84)
Q4	7.42	1.07 (0.91, 1.26)	0.65 (0.55, 0.76)	0.66 (0.56, 0.78)
Q5	8.42	1.21 (1.04, 1.40)	0.72 (0.63, 0.84)	0.68 (0.58, 0.79)
*p* for trend		<0.01	0.01	<0.01
Cardiovascular mortality				
Non-HDL-C (as continuous variables, mmol/L)	1.23	1.2 (1.09, 1.31)	1.08 (0.97, 1.21)	1.08 (0.96, 1.22)
Non-HDL-C quintiles				
Q1	0.91	Reference	Reference	Reference
Q2	1.06	1.17 (0.83, 1.63)	0.92 (0.66, 1.29)	1.00 (0.72, 1.40)
Q3	1.15	1.26 (0.90, 1.77)	0.87 (0.62, 1.23)	0.98 (0.70, 1.39)
Q4	1.46	1.60 (1.10, 2.32)	0.98 (0.69, 1.40)	1.12 (0.76, 1.65)
Q5	1.52	1.65 (1.19, 2.29)	1.04 (0.75, 1.45)	1.08 (0.75, 1.55)
*p* for trend		<0.01	0.52	0.56

Model I adjust for none. Model II adjust for age, gender, and race. Model III adjust for age, gender, race, smoking, body mass index, systolic blood pressure, family income, education level, physical activity, estimated glomerular filtration rate, comorbidities (diabetes and hypertension), and medicine use (antihypertensive drugs, hypoglycemic agents, and lipid-lowering drugs). HR, hazard ratio; CI, confidence interval; Q, quintiles.

### Dose–response relationships

**Figure 2:**
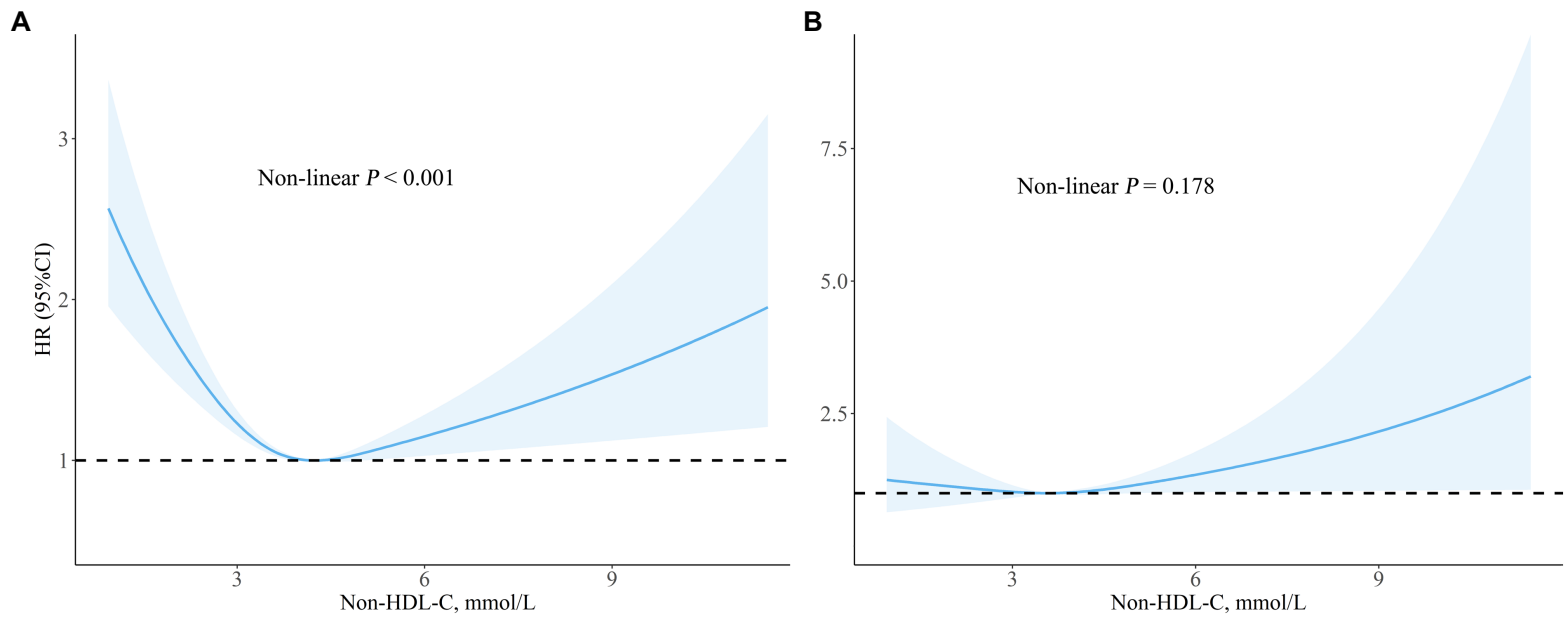
Spline analyzes of all-cause **(A)** and cardiovascular **(B)** mortality by non-HDL-C in the overall cohort (Spline analyzes were adjusted for age, gender, race, smoking, family income, education level, physical activity, body mass index, systolic blood pressure, estimated glomerular filtration rate, comorbidities (diabetes and hypertension), and medicine use (antihypertensive drugs, hypoglycemic agents, and lipid-lowering drugs.

After adjusting for confounders included in model III, the association between non-HDL-C and all-cause appeared to be U-shaped. At the same time, both low and high levels of non-HDL-C were associated with increased all-mortality risk (Figure 2). Similar relationship, but not significantly, was found between non-HDL-C and cardiovascular mortality (non-linear *p* = 0.178). The results of the regression analysis were showed in Table 3. The cut-off values for all-cause and cardiovascular mortality were 4.23 mmol/L and 3.54 mmol/L, separately. On the left of cut-off value, the HRs (95%CI) for all-cause and cardiovascular mortality were 0.78 (0.71–0.85) and 0.91 (0.69–1.19) for every 1 mmol/L increment of non-HDL-C. However, on the right side, the HRs (95%CI) for all-cause and cardiovascular mortality were 1.13 (1.00–1.27) and 1.14 (0.98–1.34), respectively.

**Table 3:** The results of two-piecewise linear regression model between non-HDL-C and cause-specific mortality.

	All-cause mortality HR (95% CI)	Cardiovascular disease mortality HR (95% CI)
Cut-off value	4.23	3.54
<Cut-off value	0.78 (0.71, 0.85)	0.91 (0.69, 1.19)
≥Cut-off value	1.13 (1.00, 1.27)	1.33 (0.98, 1.34)
*p* for log likelihood ratio test	<0.01	0.21

The two-piecewise linear regression model were adjusted for age, gender, race, smoking, family income, education level, physical activity, body mass index, systolic blood pressure, estimated glomerular filtration rate, comorbidities (diabetes and hypertension), and medicine use (antihypertensive drugs, hypoglycemic agents, and lipid-lowering drugs). HR, hazard ratio; CI, confidence interval.

### Subgroup analyzes

In Supplementary Table S1, for all-cause mortality, similar U-shaped association was detected in subgroups including participants aged <65 years old, males, non-white, not taking lipid lowering drugs, subjects with BMI < 25 kg/m^2^，highly educated population, and individuals with poverty income ratio (PIR) <2.5. While on the right side of the cut-off value of 4.23 mmol/L, for every 1 mmol/L increase, non-HDL-C elevated all-cause mortality risk significantly in individuals aged<65 years old (HR = 1.19, 95% CI: 1.05–1.36), men (HR = 1.21, 95% CI: 1.05–1.41), non-white population (HR = 1.24, 95% CI: 1.04–1.49), participants who did not taking lipid-lowering medication (HR = 1.12, 95% CI: 1.00–1.28), subjects with BMI < 25 kg/m^2^ (HR = 1.35, 95% CI: 1.14–1.59) ，highly educated population (HR = 1.35, 95% CI: 1.14–1.59), and individuals with PIR < 2.5 (HR = 1.11, 95% CI: 1.00–1.24). When considering cardiovascular mortality, U-shaped association was found only among non-white population. With each 1-SD increase in non-HDL-C level, the adjusted HR for non-white was 1.25 (95% CI, 1.01–1.54). While with each 1-SD decrease in non-HDL-C level, the adjusted HR for non-white population was 0.59 (95%CI, 0.41–0.85).

## Discussion

According to this study, non-HDL-C is associated with all-cause among general civilians. At threshold values of 4.23 mmol/L and 3.54 mmol/L, all-cause mortality and cardiovascular mortality were found to be at the lowest risk. Higher or lower non-HDL-C levels in relative terms were both related to increased mortality.

While current guidelines clarify the character of LDL-C in the development of atherosclerosis, other lipid classes, such as non-HDL-C, may be more effective predictors of CVD risk than LDL-C (14, 15). LDL-C is an important target for primary and secondary prevention. But even with maximally tolerated statins and newer lipid-lowering drugs to lower LDL-C, many patients still experienced cardiovascular events (16, 17), and statin therapy targeting serum LDL-C in patients with chronic kidney disease (CKD) did not have cardiovascular benefit or improved overall survival (18). In contrast, non-HDL-C is always the sum of the amounts of cholesterol within intermediate-density lipoproteins (IDLs), lipoprotein (a) particles, LDLs, and very low-density lipoproteins (VLDs) (19). There were several guidelines that recommended reducing non-HDL-C levels as a secondary intervention goal.

Previous studies observed positive correlations between serum non-HDL-C and cardiovascular mortality (20–25). Our main outcome included that higher non-HDL-C contributed to higher all-cause death probability, in line with earlier research findings (26–31). Non-linearity was observed between non-HDL-C and cardiovascular mortality in the spline analysis, though the test did not reach statistical significance. Such results might be attributed to the limited number of events. Similarly, Cheang et al. also found that in general population, non-HDL-C did not show significance in cardiovascular mortality, while showed a U-shape association with all-cause mortality after adjustment (32). Besides, Duncan et al. found that individuals with increased concentrations of non-HDL-C were at an elevated risk of incident atherosclerotic cardiovascular diseases (ASCVD) and deaths (26). The results are also concordant with those of Abdullah et al. who detected strong correlations between increment in non-HDL-C and death (33). And elevated non-HDL-C cholesterol was associated with increased risk of all-cause mortality and myocardial infarction (34). Our research also addressed that adults with lower non-HDL-C are exposed to higher mortality risk. Several studies revealed poorer health in patients who had lower levels of cholesterol (5, 35, 36). Additionally, according to Chiu et al., chronic kidney disease patients showed a U-shaped association with health status (6). A recent study detected a similar association between non-HDL-C and mortality among patients with hypertension (37).

In some studies, however, non-HDL-C was not connected with mortality (7, 8). Covariates or selected participants may be different in these researches, resulting in such contradictions. In order to evaluate and control for confounding, we performed subgroup analyzes according to variables that were closely related to non-HDL-C and mortality: age, gender, race, use of lipid-lowering drugs, BMI, education level, physical activity, and family income. Only certain subgroups of the study showed statistical significance for non-linear relationships. Subgroups, particularly sex stratification, would have an impact on the outcome. Non-HDL-C correlates more strongly with mortality among men than among women. Concordant with our findings, an international risk-evaluation study found male exposed to about greater chance of CVD events than female in the same range of non-HDL-C concentrations (38). This might be attributed to the estrogen induced cholesterol reduction, as well as protection of blood vessels in premenopausal female (39). It was less likely for women to die from cholesterol-related causes. However, the subgroup divided by age (≥65 years old versus <65 years old) had different results compared with previous studies (37). We did not detect similar relationship between participants aged ≥65 years old and all-cause mortality. Possible explanation for the different results could be the “harvest” phenomenon of the older individuals with hypercholesterolemia or shorter life span (38, 40). And our subgroup analyzes also demonstrated that non-HDL-C predicted more accurately in subjects not using lipid lowering drugs, which suggested that lipid-lowering drugs plays a crucial role in preventing cardiovascular risks.

Possible reasons for these results were listed below. First, there has been evidence that these lipoproteins accumulate inside the arterial intima, forming atherosclerotic plaques, which contributes to the morbidity and mortality associated with atherosclerosis (41). The serum non-HDL-C level has been positively correlated with apolipoprotein B level, a major protein on pro-atherogenic lipoproteins (42). Since LDL-C particle size inversely correlates with the serum non-HDL-C concentration (43), the relative abundance of small dense LDL particles, which is more atherogenic, may be associated with high serum levels of non-HDL-C. What’s more, guidelines suggested that non-HDL-C ≥ 220 mg/dL might indicate hereditary genetic hypercholesterolemia (14). The non-HDL-C levels in hypercholesterolemic patients are higher, making them more susceptible to ASCVD and cardiovascular death (44). On the other hand, the risk of mortality increased in the subjects with very low serum non-HDL-C levels. In the calculation formula, higher HDL-C was equal to low non-HDL-C levels, so this could be attributed either to low TC or high HDL-C levels. Genetic variation and size or function variations in HDL-C particles might explain the association between low non-HDL-C with increased mortality (45, 46). Besides, researches suggested that subjects with low serum total cholesterol levels were more likely to suffer from malnutrition and inflammation (47, 48). The effects of malnutrition are detrimental to the progression of atherosclerosis by worsening inflammation (49). Furthermore, subjects with very low serum non-HDL-C levels may also be at an increased risk of cardiovascular events due to a higher HDL-C level exhibiting altered anti-inflammatory properties. Finally, as with the obesity paradox, which is largely explained by methodological issues, including reverse causality, the U-shaped association between lipoprotein levels and mortality might also be explained by methodological issues (50). The exact mechanism of the U-shaped association still needs to be clarified by more studies.

This study examined a large cohort of participants with a relatively long median follow-up. However, potential limitations should be noted. Firstly, although extensively adjusting for many covariates, residual confounding might still exist due to unmeasured confounders such as serum levels of high-sensitivity C-reactive protein. Secondly, the blood lipids were only tested once at baseline, which might be affected by exposures that occurred after study entry. A longitudinal modeling technique approach would control for this variability, but due to aim of the current investigation, we felt that this would be beyond the scope of the present research. Besides, further analyzes are required to compare with LDL-C for the role as an effective indicator in clinical practice. Lastly, the cohort is based on the US population. Our results are supposed to be verified in different races and geographical conditions before extrapolating.

## Conclusion

Non-HDL-C was non-linearly associated with mortality among general population in a U-shaped manner in this work. Further studies are warranted in order to define the role of serum non-HDL-C as a marker for mortality risk and the optimal target range of non-HDL-C level in different population.

## Data availability statement

The datasets presented in this study can be found in online repositories. The names of the repository/repositories and accession number(s) can be found here: https://www.cdc.gov/nchs/nhanes.

## Ethics statement

The studies involving human participants were reviewed and approved by the Institutional Review Board of the Centers for Disease Control and Prevention (Protocol 98-12, 2005-06 and 2011-17). The patients/participants provided their written informed consent to participate in this study.

## Author contributions

YH and MY formed the study concept and interpreted the data. CC contributed to the data analysis. CC and YF supervised this study. YH and DZ composed and revised the manuscript. All authors contributed to the article and approved the submitted version.

## Funding

This work was supported by the Key Area R&D Program of Guangdong Province (No. 2019B020227005), the Climbing Plan of Guangdong Provincial People’s Hospital (DFJH2020022), Guangdong Provincial Clinical Research Center for Cardiovascular disease (2020B1111170011).

## Conflict of interest

The authors declare that the research was conducted in the absence of any commercial or financial relationships that could be construed as a potential conflict of interest.

## Publisher’s note

All claims expressed in this article are solely those of the authors and do not necessarily represent those of their affiliated organizations, or those of the publisher, the editors and the reviewers. Any product that may be evaluated in this article, or claim that may be made by its manufacturer, is not guaranteed or endorsed by the publisher.

## Supplementary material

The Supplementary material for this article can be found online at: https://www.frontiersin.org/articles/10.3389/fcvm.2023.1065750/full#supplementary-material
