# Supplementary material for: The U-shaped association of non-high-density lipoprotein cholesterol with all-cause and cardiovascular mortality in general adult population
Source: Front Cardiovasc Med. 2023 Feb 8;10:1065750. doi: 10.3389/fcvm.2023.1065750 (PMC9945232; doi:10.3389/fcvm.2023.1065750)
Supplement: Supplementary file 1 [file Table_1.docx]

Supplementary Table S1 Subgroups analysis

|  | All-cause mortality  HR (95% CI) | | P for log  likelihood ratio test | Cardiovascular disease mortality  HR (95% CI) | | P for log  likelihood ratio test |
| --- | --- | --- | --- | --- | --- | --- |
| Cutoff value, mmol/L | <4.23 | ≥4.23 |  | <3.54 | ≥3.54 |  |
| Age |  |  |  |  |  |  |
| >=65 | 0.82 (0.74, 0.92) | 0.94 (0.83, 1.07) | 0.18 | 1.06 (0.77, 1.45) | 1.03 (0.86, 1.22) | 0.91 |
| <65 | 0.87 (0.77, 0.99) | 1.19 (1.05, 1.36) | <0.01 | 0.86 (0.52, 1.41) | 1.19 (0.99, 1.44) | 0.23 |
| Gender |  |  |  |  |  |  |
| Male | 0.71 (0.63, 0.80) | 1.21 (1.05, 1.41) | <0.01 | 0.71 (0.50, 1.00) | 1.23 (1.02, 1.50) | 0.02 |
| Female | 0.86 (0.77, 0.97) | 0.99 (0.87, 1.12) | 0.16 | 1.30 (0.81, 2.09) | 0.98 (0.80, 1.21) | 0.38 |
| Race |  |  |  |  |  |  |
| Non-white | 0.69 (0.61, 0.78) | 1.24 (1.04, 1.49) | <0.01 | 0.59 (0.41, 0.85) | 1.25 (1.01, 1.54) | 0.01 |
| White | 0.83 (0.74, 0.92) | 1.07 (0.96, 1.19) | <0.01 | 1.15 (0.78, 1.70) | 1.08 (0.91, 1.29) | 0.78 |
| Lipid-lowering drugs |  |  |  |  |  |  |
| No | 0.75 (0.68, 0.82) | 1.13 (1.00, 1.28) | <0.01 | 0.79 (0.59, 1.07) | 1.13 (0.98, 1.30) | 0.11 |
| Yes | 0.93 (0.75, 1.14) | 1.19 (0.83, 1.70) | 0.17 | 1.23 (0.63, 2.39) | 1.36 (0.77, 2.42) | 0.79 |
| Body mass index, kg/m^2^ |  |  |  |  |  |  |
| <25 | 0.71 (0.62, 0.80) | 1.35 (1.14, 1.59) | <0.01 | 1.23 (0.79, 1.91) | 1.02 (0.76, 1.37) | 0.59 |
| ≥25 | 0.83 (0.75, 0.92) | 1.05 (0.95, 1.17) | <0.01 | 0.80 (0.56, 1.15) | 1.21 (1.01, 1.44) | 0.07 |
| Education level |  |  |  |  |  |  |
| Below high school | 0.78 (0.69, 0.88) | 1.04 (0.91, 1.19) | 0.01 | 0.58 (0.40, 0.83) | 1.17 (0.97, 1.41) | 0.01 |
| High school or above | 0.78 (0.69, 0.87) | 1.17 (1.00, 1.37) | <0.01 | 1.27 (0.86, 1.88) | 1.13 (0.90, 1.41) | 0.63 |
| Activity |  |  |  |  |  |  |
| Lower than moderate activity, % | 0.78 (0.71, 0.87) | 1.06 (0.95, 1.19) | <0.01 | 1.00 (0.69, 1.45) | 1.14 (0.96, 1.35) | 0.59 |
| Moderate activity, % | 0.80 (0.68, 0.93) | 1.14 (0.97, 1.34) | <0.01 | 0.74 (0.43, 1.26) | 1.37 (1.08, 1.73) | 0.10 |
| Vigorous activity, % | 0.76 (0.59, 0.98) | 1.22 (0.89, 1.68) | <0.01 | 1.15 (0.55, 2.41) | 0.63 (0.40, 1.01) | 0.27 |
| Poverty income ratio (PIR) |  |  |  |  |  |  |
| <2.5 | 0.80 (0.73, 0.89) | 1.11 (1.00, 1.24) | <0.01 | 0.76 (0.56, 1.03) | 1.24 (1.03, 1.48) | 0.04 |
| ≥2.5 | 0.74 (0.64, 0.86) | 1.19 (0.96, 1.47) | <0.01 | 1.16 (0.69, 1.95) | 1.01 (0.78, 1.30) | 0.65 |

When analyzing a subgroup variable, age, gender, race, smoking, family income, education level, physical activity, body mass index, systolic blood pressure, estimated glomerular filtration rate, comorbidities (diabetes and hypertension), and medicine use (antihypertensive drugs, hypoglycemic agents, and lipid-lowering drugs) were all adjusted except the variable itself.
